# A novel serosal traction technique in transcolonic endoscopic appendectomy

**DOI:** 10.1055/a-2877-1798

**Published:** 2026-06-03

**Authors:** Xiaojing Du, Shuangzhu Yang, Zehua Zhang, Meidong Xu, Haibin Zhang

**Affiliations:** 1 Endoscopy Center, Department of GastroenterologyShanghai East Hospital, Tongji University School of MedicineShanghaiChina

## Abstract

Not applicable

A 65-year-old man was admitted to our endoscopy center for evaluation of an appendiceal orifice mass detected during screening colonoscopy. He had no history of chronic diseases or surgeries. Preoperative evaluation showed that the lesion extended into the appendiceal lumen ( Fig. 1 ). Endoscopic submucosal dissection and full-thickness resection devices cannot resect ‌lesions within the appendiceal lumen, while laparoscopic appendectomy is ineffective for cecal mucosal portions. Hence, transcolonic endoscopic appendectomy (TEA) was employed for lesion resection, 1​ with a novel serosal traction technique applied to facilitate the procedure ( Video 1 ). In detail, during full-thickness resection, a small tissue of the muscularis propria and serosa‌ was preserved as a traction anchor. This was grasped by a clip with a rubber band ( Fig. 2a ). Then, the rubber band was grasped with another clip and fixed to the colon wall, thereby pulling the serosa into the colon lumen. This traction facilitates exposure of the surgical field, providing a stable operative view for the dissection of the appendiceal mesentery. After completion of the mesentery dissection, the remaining tissue was not immediately incised. Instead, a snare was employed to gently pull the appendix into the colon lumen, everting the serosa toward the lumen. This maneuver allowed easily aligning of the resection margin ( Fig. 2b ). Once the defect was essentially closed, the remaining tissue was incised and an endoloop was used to enhance the closure. The lesion was en bloc removed with appendix after a 48-minute procedure ( Fig. 3 ). The patient had no obvious complications, resumed a liquid diet on postoperative day 3, and was discharged on postoperative day 6. The results of H & E staining revealed a diagnosis of the sessile serrated lesion (SSL; Fig. 4 ).

**Fig. 1:**
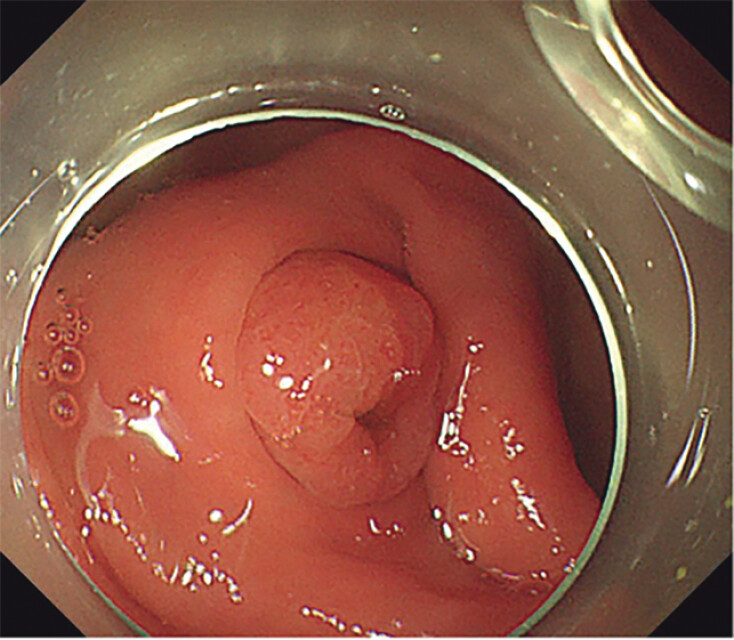
Colonoscopy showed a sessile serrated lesion at an appendiceal orifice.

**Video 1** A serosal traction technique in transcolonic endoscopic appendectomy.


**Fig. 2:**
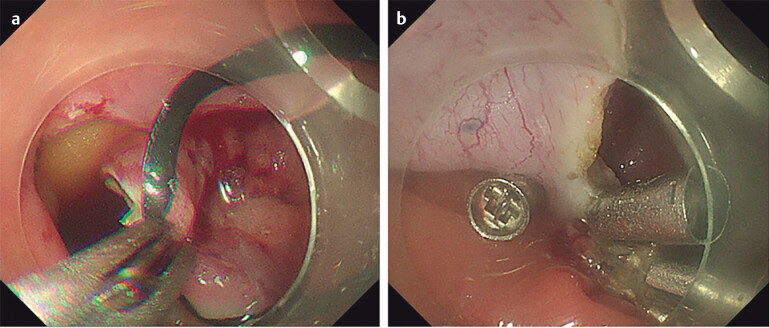
A serosal traction technique. ( **a** ) A clip with a rubber band was applied to grasp the serosa. ( **b** ) Close the defect with the assistance of serosal traction.

**Fig. 3:**
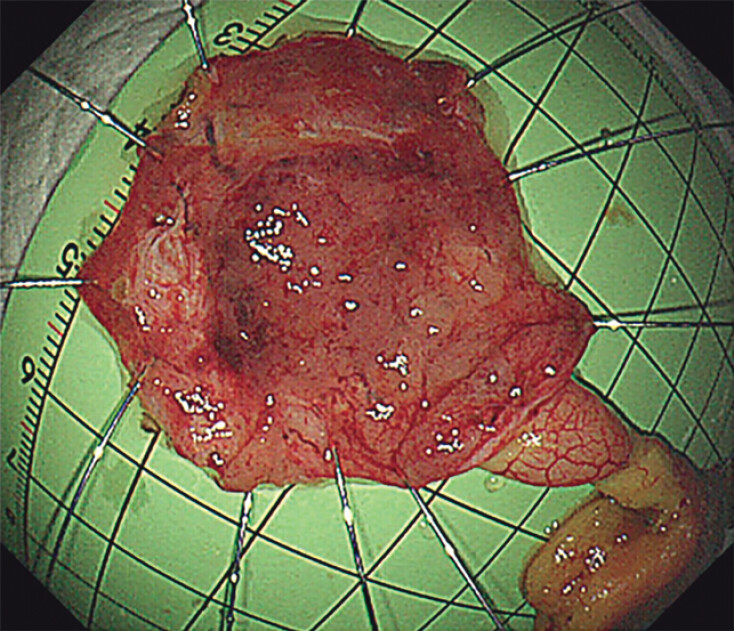
The lesion was completely removed with the appendix after a 48-minute procedure.

**Fig. 4:**
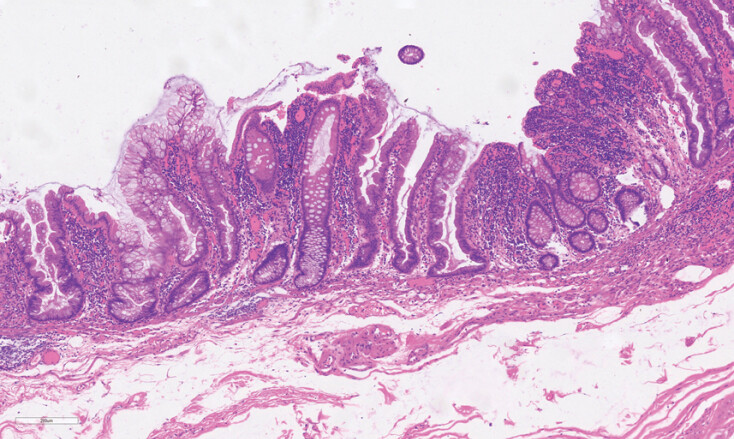
The H & E staining of lesions. The scale is 200 μm.

TEA faces two key challenges: poor surgical field visibility and difficult defect closure in deep procedures. The serosal traction technique effectively overcomes these challenges.

Endoscopy_UCTN_Code_TTT_1AO_2AG_3AF
